# GITR/GITRL reverse signalling modulates the proliferation of hepatic progenitor cells by recruiting ANXA2 to phosphorylate ERK1/2 and Akt

**DOI:** 10.1038/s41419-022-04759-z

**Published:** 2022-04-04

**Authors:** Yu He, Yufeng Pei, Kai Liu, Lin Liu, Yue Tian, Hongyi Li, Min Cong, Tianhui Liu, Hong Ma, Hong You, Jidong Jia, Dong Zhang, Ping Wang

**Affiliations:** 1 Liver Research Center, Beijing Friendship Hospital, Capital Medical University, Beijing, China; 2 Beijing Key Laboratory of Translational Medicine in Liver Cirrhosis, Beijing, China; 3 National Clinical Research Center of Digestive Disease, Beijing, China; 4 Beijing Clinical Research Institute, Beijing, China; 5 Beijing Key Laboratory of Tolerance Induction and Organ Protection in Transplantation, Beijing, China; 6 Immunology Research Center for Oral and Systemic Health, Beijing Friendship Hospital, Capital Medical University, Beijing, China; 7 General Surgery Department, Beijing Friendship Hospital, Capital Medical University, Beijing, China

**Keywords:** Cell growth, Liver fibrosis

## Abstract

Hepatic stem/progenitor cells are the major cell compartment for tissue repair when hepatocyte proliferation is compromised in chronic liver diseases, but the expansion of these cells increases the risk of carcinogenesis. Therefore, it is essential to explore the pathways restricting their expansion and abnormal transformation. The ligand of glucocorticoid-induced tumour necrosis factor receptor (GITRL) showed the most highly increased expression in hepatic progenitor cells treated with transforming growth factor (TGF)-β1. If overexpressed by hepatic progenitor cells, GITRL stimulated cell proliferation by activating the epithelial–mesenchymal transition pathway and enhancing ERK1/2 and Akt phosphorylation via GITRL binding to ANXA2. However, GITR, the specific GITRL receptor, suppressed the epithelial–mesenchymal transition pathway of GITRL-expressing cells and decreased their growth by dissociating ANXA2 from GITRL and reducing downstream ERK1/2 and Akt phosphorylation. This study identifies GITR/GITRL reverse signalling as a cross-interaction pathway between immune cells and hepatic stem/progenitor cells that restricts the expansion of hepatic stem/progenitor cells and reduces the possibility of carcinogenesis.

## Introduction

The liver is an organ with a strong regenerative capacity in response to injuries that involve hepatocytes and facultative stem/progenitor cells [1, 2]. Acute and short-term liver injuries stimulate existing hepatocytes to replicate for injury repair and cell replenishment. However, when hepatocyte proliferation is compromised in chronic liver diseases, hepatic stem/progenitor cells, resident in the portal area, serve as a major cell compartment for tissue repair and liver regeneration [3]. The number of hepatic stem/progenitor cells is increased as chronic disease progresses, and the extent of hepatocytes derived from hepatic stem/progenitor cells is correlated with disease severity [4, 5]. However, given that severe chronic liver diseases enhance the incidence of hepatocellular carcinoma (HCC) [6, 7] and that HCC shares many common markers with hepatic stem/progenitor cells [8, 9], it has been postulated that hepatic stem/progenitor cells are a possible cellular origin of HCC. Therefore, modulating the expansion or proliferation of hepatic stem/progenitor cells and preventing their abnormal transformation are essential for balancing regeneration and carcinogenesis.

Chronic hepatitis results from repeated cycles of hepatocyte death, inflammation, and regeneration. Dead hepatocytes not only activate liver resident immune cells, such as Kupffer cells, but also recruit many immune cells, including neutrophils, monocytes and T cells, to the liver tissue [10, 11]. It has become evident that the inflammatory microenvironment produced by recruited monocytes [12], macrophages [13], T cells [14, 15] and NK cells [14, 15] contributes to the expansion of hepatic stem/progenitor cells in liver injury and regeneration models. Cytokines secreted by these immune cells, including IL-22 [16], TNF [17, 18], TNF-like weak inducer of apoptosis (TWEAK) [19–21] and lymphotoxin-β [22], promote the expansion of hepatic stem/progenitor cells by binding to their specific receptor. However, in addition to tumour necrosis factor-related apoptosis-inducing ligand (TRAIL) and its receptor, which can inhibit the ductular reaction and liver fibrosis [23], cytokines with growth inhibitory effects on hepatic stem/progenitor cells require further exploration.

As an essential fibrogenic cytokine produced by Kupffer cells and liver infiltrating immune cells, transforming growth factor (TGF)-β1 enhances extracellular matrix deposition of hepatic stellate cells, which results in tissue fibrosis/cirrhosis in chronic liver diseases [24]. Upon hepatic stem/progenitor cells, TGF-β1 induces epithelial–mesenchymal transition [25], and chronic stimulation with TGF-β1 results in abnormal transformation into tumour-initiating cells, thus contributing to carcinogenesis [26].

In this study, we found that the ligand of glucocorticoid-induced tumour necrosis factor receptor (GITR), also called GITRL, showed the most significantly increased expression in hepatic progenitor cells after TGF-β1 exposure. GITRL specifically binds to GITR, which transduces a costimulatory signal in GITR-expressing immune cells [27, 28]. Since GITRL has a cytoplasmic domain, GITR binding to GITRL could transduce receptor–ligand reverse signalling to regulate the function of the GITRL-expressing cells [29, 30]. Therefore, the aim of the present study was to determine the effects of GITRL on hepatic progenitor cells and to explore GITR/GITRL reverse signalling in hepatic progenitor cells, especially focusing on proliferation, to reveal another pathway by which immune cells modulate hepatic stem/progenitor cells.

## Results

### GITRL shows the most significantly increased expression during TGF-β1-induced epithelial–mesenchymal transition of hepatic progenitor cells

**Fig. 1:**
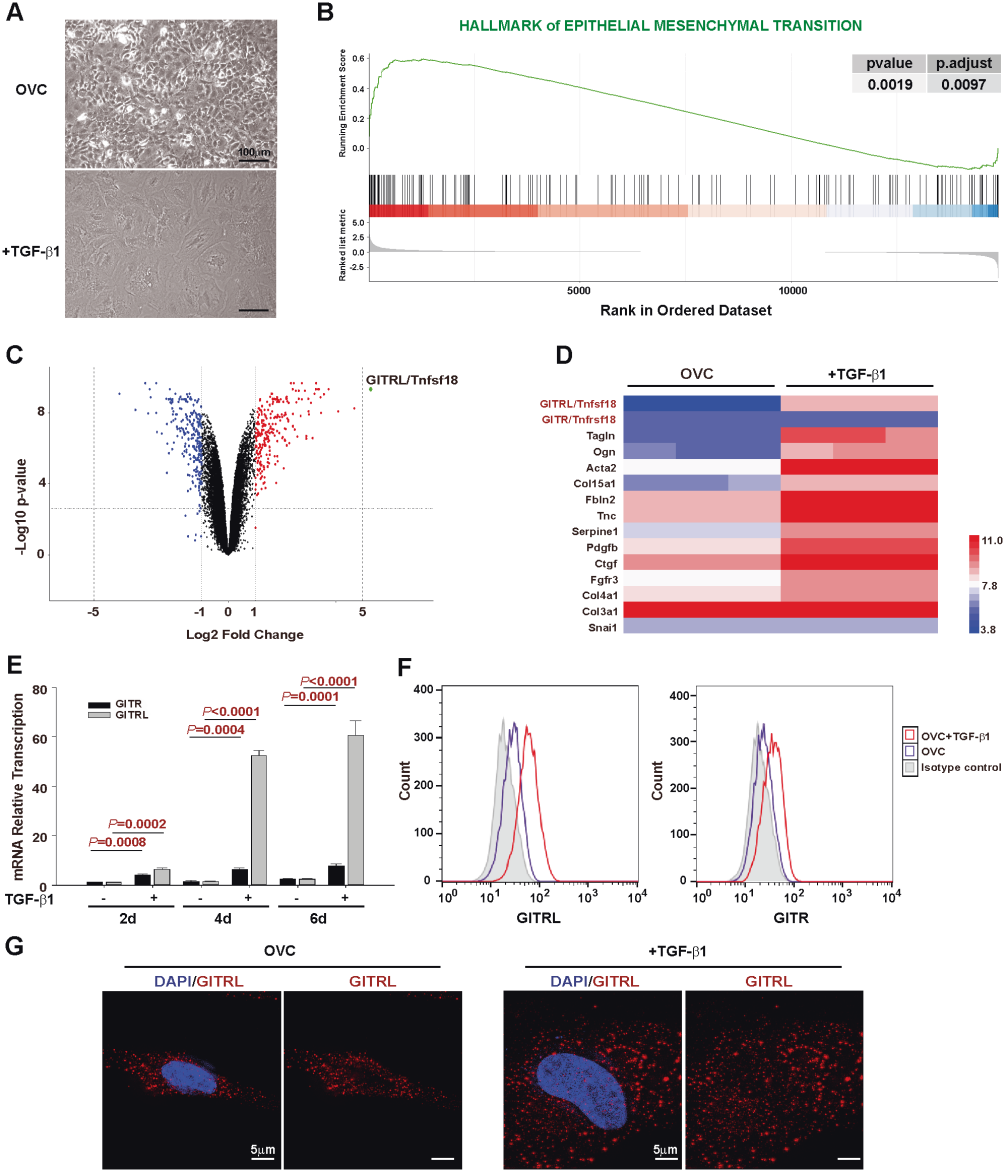
GITRL expression is significantly increased in hepatic progenitor cells experiencing epithelial–mesenchymal transition. **A** The morphology of hepatic progenitor cells and 16-day TGF-β1-treated hepatic progenitor cells. **B** RNA array and GSEA revealed activation of the hallmarks of epithelial–mesenchymal transition in 16-day TGF-β1-treated hepatic progenitor cells compared to control cells. **C** Volcano analysis of RNA array data showed that GITRL was the most significantly induced gene in hepatic progenitor cells by fold change. **D** Heatmap expression levels of GITRL, GITR, and epithelial–mesenchymal transition-related genes in the TGF-β1-treated hepatic progenitor cells and control cells by RNA arrays. **E** TaqMan RT-PCR analysis showed that TGF-β1 time-dependently induced GITRL and GITR expression in hepatic progenitor cells (*N* = 3). **F** Immunofluorescence staining without permeabilization and flow cytometry were used to analyse the expression of GITRL and GITR in the 6-day TGF-β1-treated hepatic progenitor cells. **G** 3D-SIM images of GITRL expression in the control hepatic progenitor cells and the cells treated by TGF-β1 after immunofluorescence staining without permeabilization.

Hepatic stem/progenitor cells experience epithelial–mesenchymal transition and abnormal transformation into tumour-initiating cells after chronic stimulation with the fibrogenic factor TGF-β1 [25, 26, 31]. To reveal the mechanism underlying this process, we incubated hepatic progenitor cells with TGF-β1 for 16 days (Fig. 1A), and an RNA array was used to analyse the changes in mRNA transcription. Based on the RNA array data, Gene set enrichment analyses (GSEA) revealed activation of the epithelial–mesenchymal transition pathway after TGF-β1 incubation (Fig. 1B). Among the genes with significant changes after TGF-β1 treatment, GITRL showed the most highly increased expression by fold change (Fig. 1C). Expression of GITRL and its receptor, GITR, in the heatmap of the RNA array data showed that GITRL expression increased significantly, yet GITR expression was low and had no significant changes (Fig. 1D). TaqMan RT-PCR analysis confirmed the time-dependently enhanced expression of GITRL and GITR after TGF-β1 treatment (Fig. 1E). Immunofluorescence staining without permeabilization and flow cytometry analysis showed increased expression of GITRL and slightly enhanced expression of GITR (Fig. 1F). The images of three-dimensional-Structured Illumination Microscopy (3D-SIM) (Fig. 1G and Supplementary Movies 1 and 2) showed that GITRL was expressed on the cell membrane of hepatic progenitor cells and more GITRL was expressed by TGF-β1-treated cells after immunofluorescence staining without permeabilization. As the gene with the most significantly increased expression level, GITRL may be responsible for the TGF-β1-induced epithelial–mesenchymal transition process in hepatic progenitor cells.

### Higher GITRL and GITR expression is correlated with poor clinical outcomes of HCC patients, and hepatic progenitor cells express GITRL and GITR in vivo

**Fig. 2:**
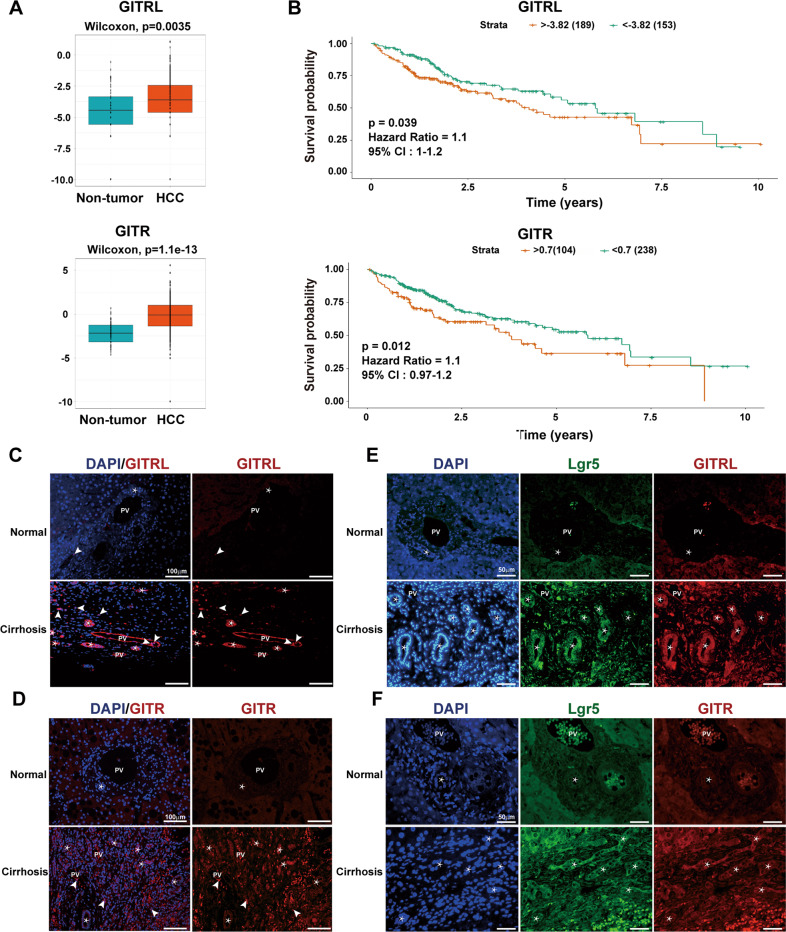
Higher GITRL and GITR expression is correlated with poor clinical outcome of HCC patients, and hepatic progenitor cells express GITRL and GITR in vivo. **A** GITRL and GITR transcription was significantly increased in HCC samples (*N* = 342) compared to nontumour samples (*N* = 50) among the LIHC data in The Cancer Genome Atlas (TCGA) clinical database. **B** Higher GITRL expression was associated with lower survival probability in the TCGA LIHC cohort with GITRL-High group *N* = 189 and GITRL-Low group N = 153. The higher the GITR expression, the lower the survival probability was in the TCGA LIHC cohort with GITRL-High group *N* = 104 and GITRL-Low group *N* = 238. **C** There were GITRL-expressing cells in one HBV-induced cirrhotic liver tissue sample, while few GITRL-expressing cells could be detected in one control human liver. **D** There were many GITR-expressing cells in one HBV-induced cirrhotic liver tissue sample, including many immune cells, while few GITR-expressing cells could be detected in one control human liver. **E** Reactive bile ductules were double positive for GITRL and Lgr5. **F** Lgr5-positive reactive bile ductules were weakly positive for GITR. Stars indicate reactive bile ductules, and the arrowhead indicates immune cells.

Since GITRL was selected by RNA microarray data, whether the expression of GITRL and its receptor is related to HCC carcinogenesis with clinical implications should be further studied. GITRL and GITR expression was analysed in LIHC samples in the TCGA database. Compared to that of nontumour tissue (*N* = 50), GITRL and GITR transcription was significantly increased in LIHC tissue (*N* = 342, *P* = 0.0035 for GITRL; *P* < 0.001 for GITR, Fig. 2A). Among the LIHC samples (*N* = 342), the overall survival of the GITRL high-expression group (*N* = 189) was significantly lower than that of the low-expression group (*N* = 153, *P* = 0.039, Fig. 2B), and that of the GITR high-expression group (*N* = 104) was also significantly lower than that of the low-expression group (*N* = 238, *P* = 0.012, Fig. 2B), suggesting the increased GITRL or GITR expression is correlated with HCC disease severity and poor clinical outcome. Furthermore, immunofluorescence analyses showed that GITRL and GITR were not expressed in the control human liver but were expressed by reactive bile ductules in hepatitis B cirrhotic human liver tissue, although GITR was weakly expressed (Fig. 2C, D). Double immunofluorescence results revealed that Lgr5 (a marker of hepatic stem/progenitor cells)-positive bile ductules co-expressed GITRL and GITR (Fig. 2E, F). Therefore, GITRL and GITR expression is increased in HCC carcinogenesis, which correlates with clinical outcome, and they are expressed by hepatic progenitor cells in chronic HBV infection-induced cirrhotic human liver tissue.

### GITRL activates the epithelial–mesenchymal transition pathway and has growth stimulatory effects on hepatic progenitor cells via ERK1/2 and Akt phosphorylation

**Fig. 3:**
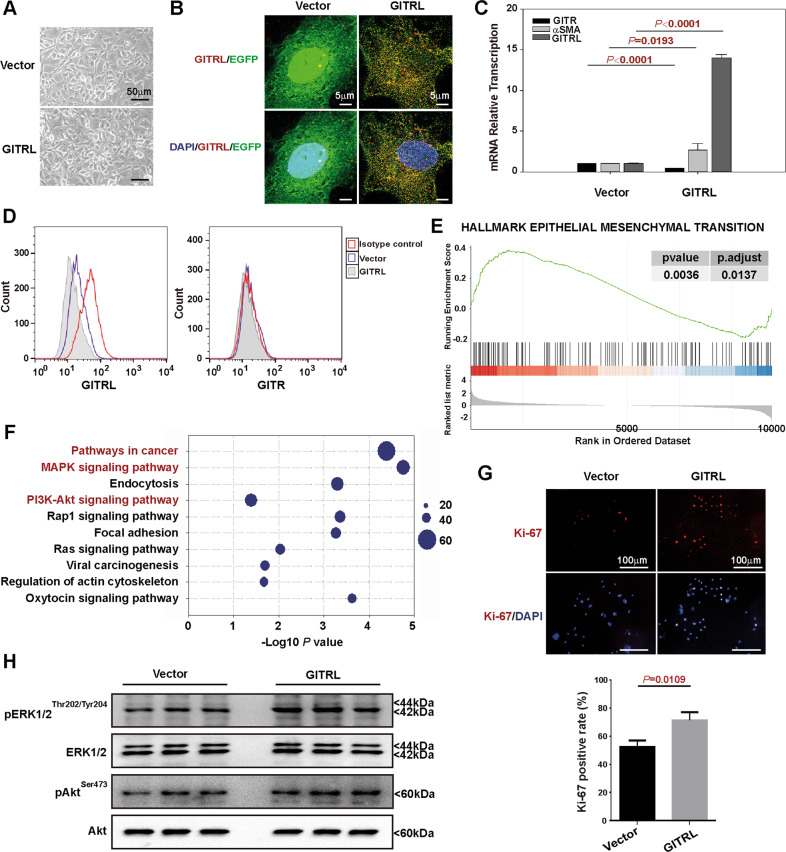
GITRL overexpression induces epithelial–mesenchymal transition and enhances the proliferation of hepatic progenitor cells via the ERK1/2 and Akt signalling pathways. **A** The morphology of GITRL-overexpressing hepatic progenitor cells and vector-transfected control cells. **B** 3D-SIM images of GITRL expression in GITRL-overexpressing hepatic progenitor cells and the vector-transfected control cells after immunofluorescence staining without permeabilization. **C** TaqMan RT-PCR results showed increased transcription of GITRL and α-SMA in the GITRL-overexpressing cells compared to the vector-transfected control cells (*N* = 3). **D** Immunofluorescence staining without permeabilization and flow cytometry showed the expression of GITRL and GITR in the GITRL-overexpressing cells. **E** GSEA based on RNA sequencing data comparing the GITRL-overexpressing cells (*N* = 3) to the vector-transfected control cells (*N* = 2) showed that overexpression of GITRL activated the epithelial–mesenchymal transition pathway. **F** Top ten pathways with significant changes after GITRL overexpression by KEGG analyses based on RNA sequencing data. **G** Overexpression of GITRL increased the Ki-67 positive rate when compared to that of the vector-transfected control cells (*N* = 3). **H** Overexpression of GITRL enhanced the phosphorylation of ERK1/2 (Thr202/Tyr204) and Akt (Ser473) compared to that of the vector-transfected control cells (*N* = 3).

Since GITRL had the most significantly increased expression after TGF-β1 treatment, GITRL was overexpressed in rat hepatic progenitor cells to determine its function (Fig. 3A). GITRL overexpression was confirmed by 3D-SIM images (Fig. 2B and Supplementary Movie 3 and 4), and TaqMan RT-PCR (Fig. 3C). When the transcription of GITR was reduced in the GITRL-overexpressing cells, the transcription of α-smooth muscle actin, a marker of mesenchymal cells, was enhanced (Fig. 3C). Immunofluorescence staining without permeabilization and flow cytometry analysis showed the enhanced expression of GITRL but no significant changes in GITR expression (Fig. 3D). RNA sequencing and GSEA comparing the GITRL-overexpressing cells to the vector-transfected control cells revealed that GITRL overexpression activated the epithelial–mesenchymal transition pathway (Fig. 3E). KEGG pathway analysis of the differentially expressed genes after GITRL overexpression revealed that pathways in cancer (59 genes) and the MAPK signalling pathway (44 genes) were the top two pathways with the highest number of significantly changed genes after GITRL overexpression (Fig. 3F). GITRL overexpression also activated 40 genes in the PI3K/Akt pathway as the fourth pathway, which had the highest number of significantly changed genes (Fig. 3F). The Ki-67-positive rate was significantly higher in the GITRL-overexpressing cells than in the vector-transfected control cells (*P* = 0.0109, Fig. 3G). Western blot analysis revealed that GITRL overexpression enhanced the phosphorylation of ERK1/2 (Thr202/Tyr204) and Akt (Ser473) in hepatic progenitor cells (Fig. 3H).

**Fig. 4:**
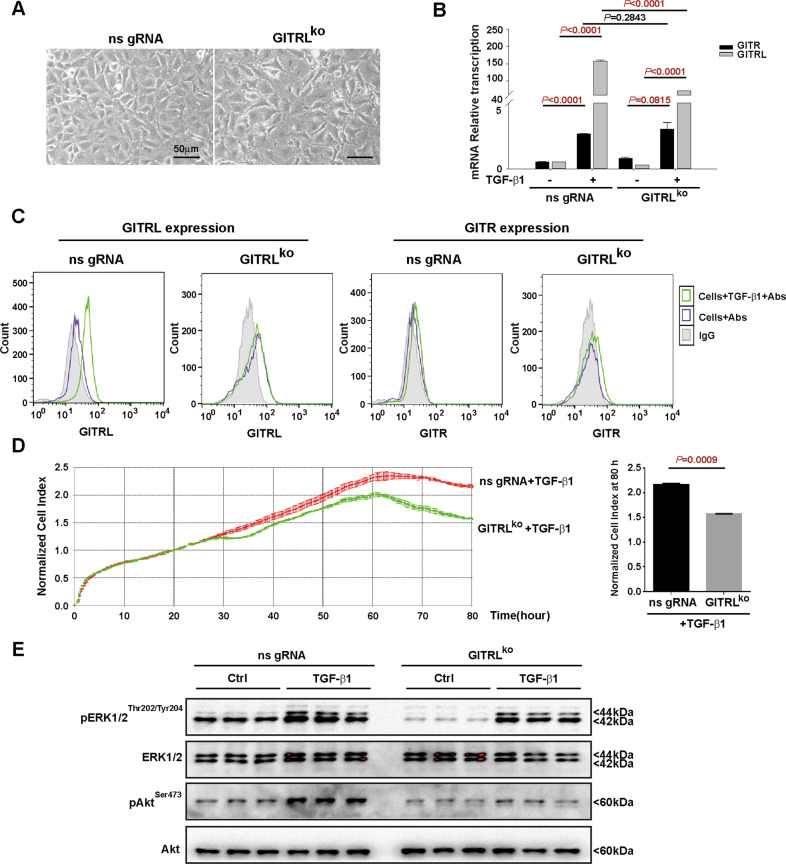
Knocking out GITRL suppresses the proliferation of hepatic progenitor cells via the ERK1/2 and Akt signalling pathways. **A** The morphology of GITRL-knockout hepatic progenitor cells and nonspecific gRNA-transfected control cells. **B** TaqMan RT-PCR results showed the transcription of GITRL and GITR in the GITRL-knockout cells compared to the nonspecific gRNA-transfected control cells in the presence of TGF-β1 (*N* = 3). **C** Immunofluorescence staining without permeabilization and flow cytometry showed the expression of GITRL and GITR in the GITRL-knockout cells after 6 days of TGF-β1 incubation. **D** xCELLigence growth curve showing that the growth of the GITRL-knockout cells (*N* = 3) was reduced compared to that of the nonspecific gRNA-transfected control cells (*N* = 3) in the presence of TGF-β1. The normalised cell index showed a significant difference at 80 h. **E** Knocking down GITRL reduced the phosphorylation of ERK1/2 (Thr202/Tyr204) and Akt (Ser473) compared to that of the nonspecific gRNA-transfected control cells regardless of the presence or absence of TGF-β1.

To confirm the growth stimulatory effects of GITRL, we knocked out GITRL by CRISPR-Cas9 in hepatic progenitor cells (Fig. 4A). TaqMan RT-PCR and flow cytometry showed that GITRL expression could not be induced by TGF-β1 in the GITRL^ko^ hepatic progenitor cells to the extent of that of the nonspecific gRNA-transfected control cells (Fig. 4B, C). Cell growth monitored by a xCELLigence growth curve showed that knocking out GITRL reduced the proliferation of hepatic progenitor cells in response to TGF-β1 compared to that of the nonspecific gRNA-transfected control cells (*P* = 0.0009) (Fig. 4D), which further confirmed the growth stimulatory effects of GITRL. Knocking out GITRL reduced ERK1/2 (Thr202/Tyr204) and Akt (Ser473) phosphorylation both in the presence and absence of TGF-β1 compared to that of the nonspecific gRNA-transfected control cells (Fig. 4E), suggesting GITRL’s contribution to ERK1/2 and Akt phosphorylation. These data suggest that GITRL induces epithelial–mesenchymal transition and has growth stimulatory effects on hepatic progenitor cells by phosphorylating ERK1/2 and Akt.

Since GITR, the receptor of GITRL, is also expressed by hepatic progenitor cells, although at a low level, GITRL may exert its growth stimulatory effects on hepatic progenitor cells by binding to GITR as described previously in CD4^+^ T cells [32] and regulatory T cells [33, 34]. To determine whether GITRL stimulates cell growth in hepatic progenitor cells by binding to GITR, we knocked out GITR by CRISPR-Cas9 in hepatic progenitor cells (Supplementary Fig. 1A). TaqMan RT-PCR analysis showed that GITR expression was significantly reduced (Supplementary Fig. 1B). The cell growth monitored by the xCELLigence growth curve revealed that knocking out GITR resulted in enhanced cell growth (*P* = 0.0001, Supplementary Fig. 1C), while GITRL knockout suppressed cell growth (*P* = 0.0002, Supplementary Fig. 1C) compared to that of the control cells in the presence of TGF-β1, suggesting different effects of GITR and GITRL on the cell growth of hepatic progenitor cells. Furthermore, knocking out GITR enhanced Akt (Ser473) phosphorylation but did not change ERK1/2 (Thr202/Tyr204) phosphorylation in the presence or absence of TGF-β1 compared to that of the nonspecific gRNA-transfected control cells (Supplementary Fig. 1D), suggesting that GITR inhibits cell growth by reducing Akt phosphorylation. When recombinant GITRL (2 μg/ml) was added to the control or TGF-β1-treated cells, cell growth monitored by the xCELLigence growth curve showed GITRL did not change cell growth of control cells (*P* = 0.4697, Supplementary Fig. 1E), but further reduced cell growth of the TGF-β1-treated cells (*P* = 0.0311, Supplementary Fig. 1E), which does not support the notion that GITRL stimulates cell growth of hepatic progenitor cells by binding to GITR.

### GITR has growth inhibitory effects on the GITRL-expressing hepatic progenitor cells

**Fig. 5:**
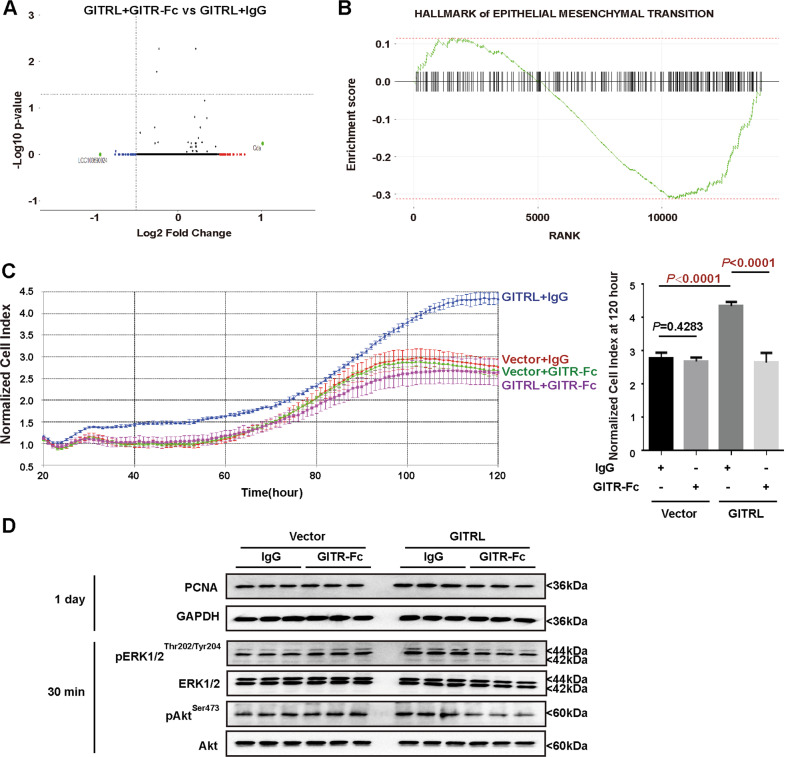
GITR inhibits the proliferation of GITRL-overexpressing hepatic progenitor cells. **A** Volcano analysis of RNA sequencing data showed that GITR incubation resulted in few genes with significant transcriptional changes. **B** Fast GSEA showed that GITR incubation induced a reduction in the hallmarks of epithelial–mesenchymal transition. **C** The xCELLigence growth curve showed that the growth of the GITRL-overexpressing cells was accelerated compared to that of the vector-transfected control cells, while GITR-Fc significantly inhibited the growth of hepatic progenitor cells (*N* = 4). The normalised cell index showed a significant difference at 120 h. **D** GITRL overexpression increased the expression of PCNA and the phosphorylation of ERK1/2 (Thr202/Tyr204) and Akt (Ser473) compared to that of the vector-transfected control cells, whereas GITR-Fc reduced the expression of PCNA and the phosphorylation of ERK1/2 (Thr202/Tyr204) and Akt (Ser473) in the GITRL-overexpressing cells (*N* = 3).

**Fig. 6:**
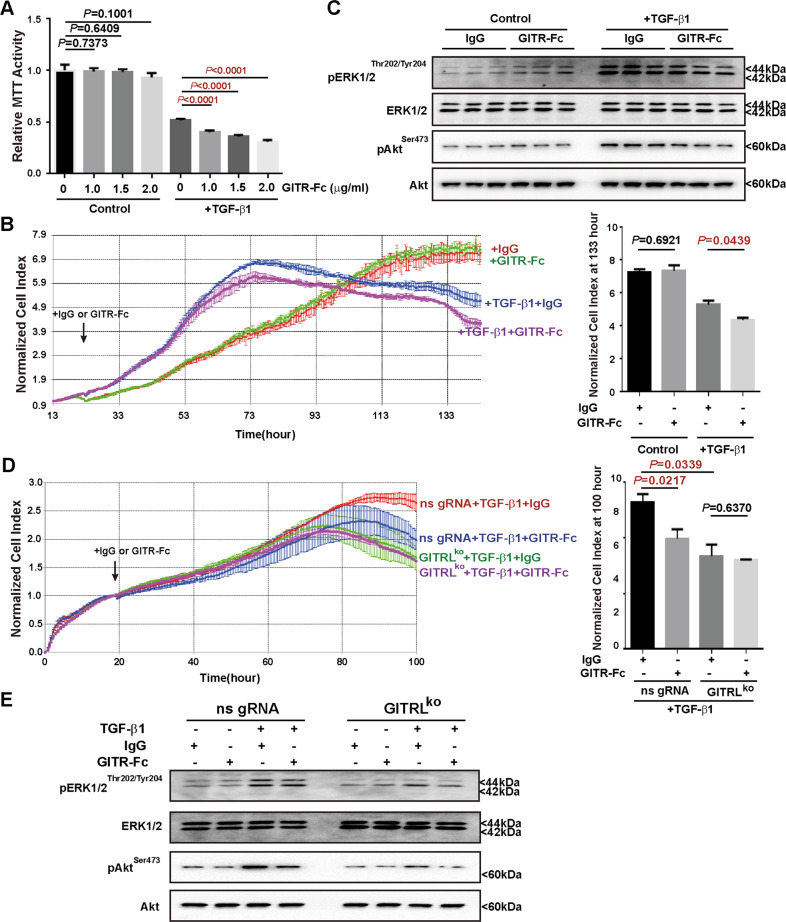
GITR inhibits the proliferation of TGF-β1-induced GITRL-expressing hepatic progenitor cells. **A** GITRL expression was induced by treatment with TGF-β1 for 6 days in hepatic progenitor cells. GITR-Fc showed a dose-dependent suppression of cell viability of the GITRL-expressing cells, as demonstrated by MTT assays, but did not inhibit the growth of the control cells (*N* = 4). **B** Cell growth was monitored and recorded every 1 h for 140 h by the xCELLigence growth curve, showing that GITR-Fc had no effects on the growth of control hepatic progenitor cells but significantly inhibited the growth of the TGF-β1-pretreated hepatic progenitor cells (*N* = 3). The normalised cell index showed a significant difference at 133 h. **C** TGF-β1 treatment enhanced the phosphorylation of ERK1/2 (Thr202/Tyr204) and Akt (Ser473) in the hepatic progenitor cells compared to the nontreated control cells, while GITR-Fc reduced the phosphorylation of ERK1/2 (Thr202/Tyr204) and Akt (Ser473) in the TGF-β1-treated cells (*N* = 3). **D** The xCELLigence growth curve showed that GITR-Fc inhibited the growth of the TGF-β1-treated nonspecific gRNA-transfected control cells but had no effects on the growth of the TGF-β1-treated GITRL-knockout cells (*N* = 3). The normalised cell index showed a significant difference at 100 h. **E** Knocking out GITRL reduced ERK1/2 (Thr202/Tyr204) and Akt (Ser473) phosphorylation, and the GITR-Fc-medicated reduction of ERK1/2 (Thr202/Tyr204) and Akt (Ser473) phosphorylation was not as significant as TGF-β1-treated nonspecific gRNA-transfected control cells.

Since GITRL has a cytoplasmic domain, the binding of GITR to GITRL may transduce reverse signalling in GITRL-expressing cells [29]. We investigated in whether GITR expressed by immune cells affects GITRL-overexpressing hepatic progenitor cells. Recombinant rat GITR with mouse IgG2A Fc chimaera (2 μg/ml) or mouse IgG2A Fc (2 μg/ml) was used to treat the GITRL-overexpressing cells for 2 days. RNA sequencing data showed little gene transcription variation with a log2-fold change over 1 or less than 1 after GITR-Fc treatment (Fig. 5A), suggesting that GITR to GITRL reverse signalling has little impact on the gene transcription of the GITRL-overexpressing hepatic progenitor cells. Fast GSEA (Fig. 5B) revealed that GITR-Fc suppressed the epithelial–mesenchymal transition pathway of the GITRL-overexpressing cells. Cell growth monitored by the xCELLigence growth curve showed that although GITRL overexpression accelerated cell proliferation, GITR-Fc (2 μg/ml) significantly suppressed the proliferation of the GITRL-overexpressing cells (*P* < 0.001, Fig. 5C). The reduced level of PCNA expression also supported the growth inhibitory effects of GITR on the GITRL-overexpressing cells (Fig. 5D). Moreover, GITR-Fc (2 μg/ml) reduced the phosphorylation of ERK1/2 (Thr202/Tyr204) and Akt (Ser473) in the GITRL-overexpressing hepatic progenitor cells (Fig. 5D), indicating that GITR-Fc has growth inhibitory effects on the GITRL-overexpressing cells by reducing the phosphorylation of ERK1/2 and Akt. To determine whether GITR-Fc has similar effects on the TGF-β1-induced GITRL-expressing cells, we treated hepatic progenitor cells with TGF-β1 for 6 days to induce GITRL expression (Fig. 1E, F), and different doses (0.5, 1, 1.5 or 2 μg/ml) of GITR-Fc were incubated with the TGF-β1-treated cells. Cell viability revealed by MTT assays showed that GITR-Fc had little effect on the control hepatic progenitor cells but dose-dependently reduced the viability of the TGF-β1-treated cells (Fig. 6A). Cell growth monitored by the xCELLigence growth curve was consistent with the cell viability data of the MTT assays and showed that GITR-Fc (2 μg/ml) suppressed the growth of the TGF-β1-treated cells (*P* = 0.0439) but had no effects on the control cells (*P* = 0.6921, Fig. 6B). Western blot analysis showed that the TGF-β1-treated cells had higher phosphorylation levels of ERK1/2 (Thr202/Tyr204) and Akt (Ser473) than the control cells (Fig. 6C). GITR-Fc (2 μg/ml) reduced the phosphorylation levels of ERK1/2 (Thr202/Tyr204) and Akt (Ser473) in the TGF-β1-treated cells but had little effect on the control cells (Fig. 6C), suggesting that GITR-Fc suppresses the growth of the TGF-β1-induced GITRL-expressing cells by reducing ERK1/2 and Akt phosphorylation.

To confirm the effects of GITR-Fc on GITRL, we used GITR-Fc to treat the GITRL^ko^ hepatic progenitor cells. GITR-Fc (2 μg/ml) inhibited the growth of the TGF-β1-treated nonspecific gRNA-transfected control cells (*P* = 0.0217) but had no such effects on the TGF-β1-treated GITRL^ko^ cells (*P* = 0.6370, Fig. 6F), thus confirming that GITR-Fc exerts growth inhibitory effects via GITRL. Mechanistically, the GITR-Fc-mediated reduction of ERK1/2(Thr202/Tyr204) and Akt(Ser473) phosphorylation in the TGF-β1-treated GITRL-knockout cells was not as substantial as that in the TGF-β1-treated nonspecific gRNA-transfected control cells (Fig. 6F), indicating that GITR/GITRL reverse signalling inhibits cell growth of hepatic progenitor cells by reducing ERK1/2 and Akt phosphorylation.

### Annexin A2 (ANXA2) controls GITR/GITRL reverse signalling in hepatic progenitor cells

**Fig. 7:**
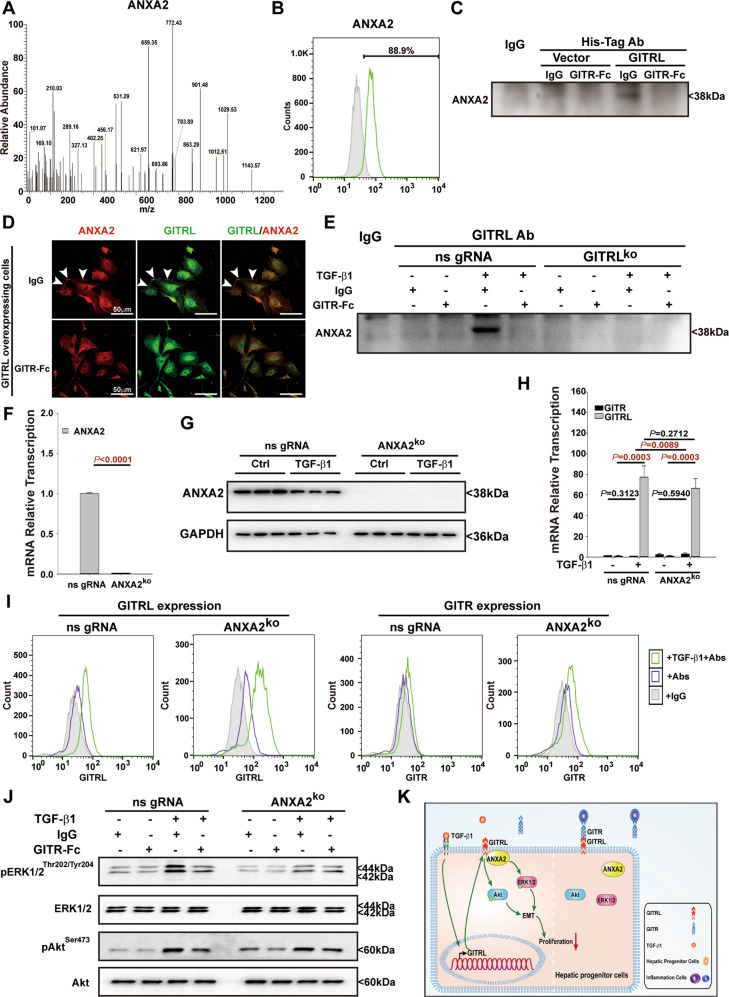
GITR/GITRL reverse signalling is dependent on recruiting ANXA2. **A** His-tag antibodies were used to co-IP GITRL binding proteins in the GITRL-overexpressing cells, and mass spectrometry analysis revealed that ANXA2 was a protein bound to GITRL. **B** Hepatic progenitor cells expressed ANXA2 at a positive rate of 88.9 ± 2.4% (*N* = 3). **C** Western blots of the proteins pulled down by His-tag antibodies showed that GITRL bound to ANXA2 in the GITRL-overexpressing cells, but GITRL dissociated from ANXA2 in the presence of GITR-Fc. **D** Double immunofluorescence staining of ANXA2 and GITRL showed that GITRL colocalized with ANXA2 on the cell membrane in the GITRL-overexpressing cells, while no colocalization was found in the presence of GITR-Fc (*N* = 3). **E** Western blots of the proteins pulled down by GITRL antibodies showed that GITRL bound to ANXA2 in the TGF-β1-treated hepatic progenitor cells, but few ANXA2 bound to GITRL in the presence of GITR-Fc. In the TGF-β1-treated GITRL-knockout cells, ANXA2 could not be pulled down by GITRL antibodies. **F** TaqMan RT-PCR results showed reduced transcription of ANXA2 (*N* = 3). **G** Western blot results showed that ANXA2 expression was knocked out in the ANXA2 knockout cells (*N* = 3). **H** TaqMan RT-PCR results showed the transcription of GITRL and GITR in the ANXA2 knockout cells compared to the nonspecific gRNA-transfected control cells in response to TGF-β1 incubation (*N* = 3). **I** Immunofluorescence staining without permeabilization and flow cytometry showed the expression of GITRL and GITR in the ANXA2 knockout cells. **J** Knocking out ANXA2 inhibited ERK1/2 (Thr202/Tyr204) and Akt (Ser473) phosphorylation, and GITR-Fc could not reduce ERK1/2 (Thr202/Tyr204) and Akt (Ser473) phosphorylation in the presence of TGF-β1 compared to that of the nonspecific gRNA-transfected control cells (*N* = 3). **K** Schematic representation of the molecular mechanism of GITR/GITRL reverse signalling in hepatic progenitor cells.

To further reveal the underlying mechanism of GITR/GITRL reverse signalling in hepatic progenitor cells, we used His-tagged antibodies to coimmunoprecipitate GITRL binding proteins in the GITRL-overexpressing cells based on the His-tag added to the GITRL overexpression plasmid. Mass Spec analysis revealed that ANXA2 was one of the proteins that bound to GITRL (Fig. 7A). Immunofluorescence staining and flow cytometry showed that hepatic progenitor cells highly expressed ANXA2 (Fig. 7B). Coimmunoprecipitation and western blot analysis confirmed the binding of ANXA2 and GITRL in the GITRL-overexpressing hepatic progenitor cells, but GITRL could no longer bind to ANXA2 in the presence of GITR-Fc (Fig. 7C). Double immunofluorescence staining data showed that GITRL colocalized with ANXA2 on the cell membrane in the GITRL-overexpressing cells, while no colocalization signal on the cell membrane could be detected in the GITR-Fc-treated GITRL-overexpressing cells (Fig. 7D), indicating that the binding of GITR to GITRL will result in dissociation of ANXA2 and GITRL. Furthermore, coimmunoprecipitation and western blot analysis showed that GITRL bound to ANXA2 in the TGF-β1-treated nonspecific gRNA-transfected control cells, while GITRL could not bind to ANXA2 after GITRL knockout or GITR-Fc incubation (Fig. 7E), further confirming the binding of GITRL to ANXA2 and the binding of GITR to GITRL for dissociating ANXA2 from GITRL.

To confirm that ANXA2 is responsible for GITR/GITRL reverse signalling, we knocked out ANXA2 by CRISPR-Cas9 in hepatic progenitor cells (Fig. 6F, G). TaqMan RT-PCR and flow cytometry revealed that knocking out ANXA2 had little impact on TGF-β1-induced GITRL expression (Fig. 6H, I). However, the phosphorylation of ERK1/2 (Thr202/Tyr204) and Akt (Ser473) was reduced in the ANXA2^ko^ cells in the presence of TGF-β1 compared to that of the nonspecific gRNA-transfected control cells (Fig. 6J), suggesting that GITRL could not enhance ERK1/2 (Thr202/Tyr204) and Akt (Ser473) phosphorylation without ANXA2. When GITR-Fc (2 μg/ml) was added to the TGF-β1-treated ANXA2^ko^ cells, the reductions in ERK1/2 (Thr202/Tyr204) and Akt (Ser473) phosphorylation were not as strong as those in the TGF-β1-treated nonspecific gRNA-transfected control cells (Fig. 6J), suggesting that GITR/GITRL reverse signalling modulates the proliferation of hepatic progenitor cells by recruiting ANXA2 to phosphorylate downstream ERK1/2 and Akt (Fig. 6K).

## Discussion

This study provides two new findings for understanding the molecular mechanism of GITR/GITRL reverse signalling in the expansion of hepatic progenitor cells. First, we found that GITRL, expressed by hepatic progenitor cells, has growth stimulatory effects by binding to ANXA2 and activating downstream ERK1/2 and Akt phosphorylation. Second, we found that when GITR, the GITRL receptor expressed by of immune cells, binds to GITRL, it transduces a receptor–ligand reversing signalling to suppress the growth of hepatic progenitor cells by dissociating ANXA2 from GITRL, thus reducing ERK1/2 and Akt phosphorylation.

Hepatic stem/progenitor cells expand in an immune cell-mediated inflammatory microenvironment. There exists a close interaction between these two kinds of cells: cytokines (such as TNF, IL-6, TWEAK and IL-22) secreted by immune cells modulate the behaviour of hepatic stem/progenitor cells [10, 11], and simultaneously, cytokines (including CCl2) expressed by hepatic stem/progenitor cells impact immune cells [35]. All these cytokines exert a ligand-to-receptor signal on the cells expressing the receptors. In this study, we reveal a molecule, GITRL, which may not only serve as a ligand to modulate the function of receptor-expressing immune cells but also transduces signals to affect ligand-expressing hepatic progenitor cells themselves.

GITRL has a growth stimulatory effect on hepatic progenitor cells. Previously, GITRL was reported to be expressed by endothelial cells and antigen-presenting cells in response to glucocorticoids [28, 36], and IFNα and IFNβ could enhance its expression in endothelial cells [37]. We found that the fibrogenic factor TGF-β1 could induce hepatic progenitor cells to express GITRL, hepatic progenitor cells are another cell compartment expressing GITRL in chronic HBV-induced cirrhotic human liver tissue, and high GITRL expression correlates with disease severity and poor clinical outcome of HCC. Our overexpression and silencing data support the growth stimulatory effect of GITRL on hepatic progenitor cells, which is similar to the effect of GITRL on macrophages [38]. As a cell compartment involved in HCC carcinogenesis [8, 9], hepatic progenitor cells expressing GITRL activate pathways of cancer, MAPK, PI3K/Akt and epithelial–mesenchymal transition by binding to ANXA2, which could enhance the progression of HCC [39–41] and many other tumours, including colorectal cancer [42], pancreatic cancer [43], breast cancer [44, 45] and prostate cancer [46], suggesting that GITRL may contribute to the abnormal transformation of hepatic progenitor cells.

As the specific receptor of GITRL, GITR has cell type-specific functions after binding to GITRL. GITR activates effector and regulatory T cells and enhances their proliferation by engagement of GITRL [32–34]. However, GITR inhibits NK cell proliferation in GITR-deficient mice with intestinal inflammation [47], and stimulation of GITR by its agonist antibodies suppresses the proliferation of NK cells by lowering stat5 and Akt phosphorylation [48]. Similar to NK cells, our data showed that GITR has growth inhibitory effects on hepatic progenitor cells, and activation of GITR by GITRL further suppresses cell growth. So, our data do support the notion that ligand-to-receptor signalling of GITRL/GITR is responsible for the growth stimulatory effects of GITRL.

When binding to GITR, GITRL transduces receptor-to-ligand reverse signalling to modulate the function of GITRL-expressing cells [29, 30]. For endothelial cells, reverse signalling through GITRL promotes phosphorylation of STAT1a and STAT1b to increase the expression of intercellular adhesion molecule-1 (ICAM-1) and vascular cell adhesion molecule-1 (VCAM-1) for immune cell adhesion [30]. For antigen-presenting cells, reverse signalling through GITRL activates NF-κB-dependent expression of indoleamine 2,3-dioxygenase in dendritic cells [29] and enhances ERK1/2/NF-κB-dependent expression of matrix metalloproteinase-9 and intercellular adhesion molecule-1 in macrophages [49]. In contrast to the growth stimulatory signals of GITR on GITRL to increase STAT or ERK1/2 phosphorylation, we found that GITR exerts growth inhibitory effects on the GITRL-expressing hepatic progenitor cells by reducing ERK1/2 and Akt phosphorylation. The growth inhibitory effects of GITR are supported by the finding that GITR reduces the number of neural stem/progenitor cells and induces their apoptosis in a murine cortical infarction model [50]. The effects of GITR/GITRL reverse signalling on hepatic stem/progenitor cells in vivo still need further exploration.

Thus, GITRL has growth stimulatory effects on hepatic progenitor cells by recruiting ANXA2 to phosphorylate ERK1/2 and Akt. GITR expressed by immune cells exerts growth inhibitory effects on hepatic progenitor cells by dissociating ANXA2 from GITRL, thus revealing a modulation pathway for immune cells to restrict the expansion of hepatic stem/progenitor cells and reduce the possibility of carcinogenesis.

## Methods

### Cell culture and stable cell line generation

Hepatic progenitor cells were isolated by collagenase perfusion and discontinuous gradient centrifugation from male Sprague-Dawley rats (130–150 g) fed a CDE diet and cultured in DMEM/F12 medium containing 10% foetal bovine serum (FBS, Gibco, Grand Island, NY, USA), epidermal growth factor (EGF, PeProTech, Rehovot, Israel), and stem cell factor (SCF, PeProTech) as described previously [51]. For TGF-β1 treatment, the cells were treated with 1 ng/ml TGF-β1 (PeProTech) in DMEM/F12 medium containing 10% FBS. For GITR-Fc treatment, the cells were treated with 0.5, 1, 1.5 or 2 μg/ml recombinant rat GITR with mouse IgG2A Fc chimaera (R&D Systems, Minneapolis, MN, USA) or with recombinant mouse IgG2A Fc Protein (R&D Systems) used as an IgG control. For GITRL treatment, the cells were treated with 2 μg/ml recombinant rat GITRL (R&D Systems).

For the generation of GITRL-overexpressing cells, a total of 3 × 10^5^ hepatic progenitor cells per well were plated in a six-well plate, and the cells were transfected with 2.5 μg rat GITRL-EGFP/N1 plasmids [Beijing Genomics Institute (BGI), Beijing, China] or EGFP/N1 vector (BD Biosciences Clontech, Palo Alto, CA, USA) using Lipofectamine 3000 (Life Technologies, Carlsbad, CA, USA) according to the manufacturer’s instructions. Four days post transfection, the cells were passaged to 60-mm plates and cultured in the presence of G418 antibiotic selection at 200 µg/ml for 14 days, thus obtaining GITRL-overexpressing hepatic progenitor cells and vector control cells.

GITRL or GITR or ANXA2 knockout hepatic progenitor cells were prepared by a two-step CRISPR-Cas9 system. First, 3 × 10^5^ hepatic progenitor cells per well were plated in a six-well plate, and the cells were infected with lentivirus containing the Cas9 gene (MOI = 10, SyngenTech, Beijing, China). Four days post infection, the cells were passaged to 60-mm plates and cultured in the presence of blasticidin at 4 µg/ml for 7 days, thus obtaining Cas9-expressing hepatic progenitor cells. Second, 3 × 10^5^ Cas9-expressing hepatic progenitor cells were plated in a 6-well plate, and the cells were transfected with 2.5 μg rat GITRL gRNA (GGTTAGAACTCATTCTCTGG) plasmids (SyngenTech), rat GITR gRNA (CAGTGCGTTGACTGTGCCAT) plasmids (SyngenTech), rat ANXA2 gRNA (TCGCCTACCAGAGAAGGACC) plasmids (SyngenTech), or rat nonspecific gRNA (ns gRNA) plasmids (SyngenTech) using Lipofectamine 3000 (Life Technologies) according to the manufacturer’s instructions. Four days post transfection, the cells were passaged to 60-mm plates and cultured in the presence of puromycin at 6 µg/mL for 7 days, and single clones were selected for further identification and expansion.

### Transcriptional analysis by TaqMan RT-PCR

Cells (2 × 10^6^) were used for total RNA extraction, reverse transcription, and TaqMan real-time quantitative polymerase chain reaction (RT-PCR). Reverse transcription was carried out with dT-primed Script II Reverse Transcriptase (Life Technologies). qPCR analyses were carried out with triplicates of each sample cDNA on an ABI7500 Fast Real-Time PCR System (Applied Biosystems, Foster City, CA, USA) with TaqMan Fast Advanced Master Mix (Applied Biosystems) and TaqMan gene expression assay for detection of GITRL, GITR, αSMA, ANXA2, and GAPDH (Supplementary Table S1, Applied Biosystems).

### GITRL and GITR expression in human liver tissue

The 421 primary liver hepatocellular carcinoma (LIHC) samples in The Cancer Genome Atlas (TCGA) clinical data, including RNA sequencing (RNA-seq) data and clinical data, were obtained using the R/Bioconductor package [52]. Among the 421 LIHC samples, there were 371 HCC samples and 50 nontumour tissues. Two cases of non-HCC liver cancer were excluded, and 27 cases were excluded due to lack of GITRL expression. mRNA expression levels were normalised, and GITRL or GITR mRNA expression was compared between cancerous (*N* = 342) and nontumour tissues (*N* = 50). In cancerous samples (*N* = 342), patients were best separated into two groups (GITRL-High and GITRL-Low or GITR-High and GITR-Low) based on minprop = 0.3 by the R/Survminer package. The hazard ratio, 95% CI, and significance of the difference were calculated by the R/survival package.

One donor liver tissue unsuitable for transplantation and one cirrhotic liver tissue with paraffin sections were used for immunofluorescence staining with clinicopathological characteristics included in Supplementary Table S2. The tissues were obtained from the Clinical Data and Biobank Resource of Beijing Friendship Hospital with the approval of the Ethics Committee of Beijing Friendship Hospital, Capital Medical University (No. 2018-P2-055-01, Beijing, China).

### Immunofluorescence staining

Paraffin tissue sections were used for standard immunofluorescence staining to detect the expression of GITRL (Invitrogen, Carlsbad, CA, USA), Lgr5 (OriGene, Rockville, MD, USA) or ANXA2 (Cell Signaling Technology, Danvers, MA, USA) as described previously [53]. Sections were examined under a Nikon 50i fluorescence microscope (Nikon, Japan).

For immunofluorescence analysis, the cultured cells were fixed with 4% paraformaldehyde and stained with GITRL antibodies (ProteinTech, Rosemont, IL) by standard immunofluorescence staining protocol without permeabilization. Images of 3D-SIM were acquired on the DeltaVision OMX V3 imaging system (Cytiva, GE Healthcare) with a ×100/1.40 NA oil objective (Olympus UPlanSApo), solid-state multimode lasers (488, 405 nm, 561 nm) and electron-multiplying CCD (charge-coupled device) cameras (Evolve 512 × 512, Photometrics). Serial Z-stack sectioning was done at 125 nm of intervals and tacks of SIM images were reconstructed using softWoRx 6.1.1 (Cytiva, GE Healthcare).

For immune-colocalization analysis, the cultured cells were fixed with 4% paraformaldehyde and permeabilized for standard intracellular immunofluorescence staining as described previously [54] to detect GITRL (ProteinTech) and ANXA2 (Cell Signaling Technology) by a Nikon A1 confocal microscope (Nikon).

For flow cytometry analysis, the cultured cells were digested by trypsin and fixed with 4% paraformaldehyde for surface immunofluorescence staining without permeabilization to detect the expression of GITRL (ProteinTech) and GITR (Abcam, Cambridge, UK) or for standard intracellular immunofluorescence staining to detect the expression of ANXA2 (Cell Signaling Technology) by FACSCalibur flow cytometry (BD Biosciences) using CellQuest software (BD Biosciences) as described previously [51].

### RNA sequencing and data analysis

RNA sequencing and library preparation of IgG-treated GITRL-overexpressing cells (*N* = 2) vs. IgG-treated vector-transfected control cells (*N* = 2) or GITR-Fc- (*N* = 3) vs. IgG (*N* = 3)-treated GITRL-overexpressing cells were performed by BGI as described previously [54]. Sequencing data along with the study design have been submitted to the NCBI Sequence Read Archive and are available under study accession number PRJNA698431 (https://dataview.ncbi.nlm.nih.gov/object/PRJNA698431). Differential expression analysis was performed using differential *P* values < 0.05 and log2(fold change) >1.0 or < −1.0. Pathway analysis was determined by mapping differentially expressed genes onto https://david-d.ncifcrf.gov/for functional annotation for signalling pathway analysis.

### GSEA for RNA array or RNA sequencing data

GSEA was performed by the R/clusterProfiler package by annotating rat gene sets to predefined human gene sets from the Molecular Signatures Database [55]. A list of ranked genes from RNA array data with GEO accession number GSE165858 was used for GSEA to compare the TGF-β1-treated cells to the control cells [56]. A list of ranked genes from the RNA sequencing data of PRJNA698431 was used for fast GSEA performed by the R/Bioconductor package to compare the IgG-treated GITRL-overexpressing cells to the IgG-treated vector-transfected control cells [57].

### Cell growth and proliferation analysis

Cell growth and proliferation were analysed by growth curves and the Ki-67-positive rate as described previously [54]. For growth curves, a total of 3 × 10^3^ cells were plated in triplicate in antibiotic-free complete medium in E-Plate 16 (ACEA Biosciences, San Diego, CA, USA) on the xCELLigence Real-Time Cell Analyzer (RTCA)-MP system (ACEA Biosciences) according to the manufacturer’s instructions. The cell index (CI) was read automatically and continuously recorded every hour as CI ± SD. Growth stimulation was determined at the time of maximum cell index (CImax), and a non-parametric *t* test was used to analyse the significant differences in cell growth. For Ki-67-positive rate analysis, the investigator was blinded to the samples, and ImageJ 1.51j8 (National Institute of Health, USA) was used for analysis.

### Western blot

Protein extracts were prepared and analysed by western blots according to standard protocols as described previously [51] using primary antibodies, including ERK1/2 (1:2000, Cell Signaling Technology), phospho-p44/42 MAPK [ERK1/2(Thr202/Tyr204), 1:2000, Cell Signaling Technology], Akt (1:2000, Cell Signaling Technology), phosphor-Akt(Ser473) (1:2000, Cell Signaling Technology), and ANXA2 (1:2000, Cell Signaling Technology). Bands were detected using the Molecular Imager ChemiDoc XRS + with Image Lab Software version 3.0 (Bio-Rad, Hercules, CA, USA).

### Immunoprecipitation (IP) and mass spectrometry analysis

Immunoprecipitation was carried out according to the manufacturer’s instructions of the Pierce^TM^ MS-compatible Magnetic IP kit (Protein A/G, Thermo, Rockford, IL, USA). Briefly, 1.0 × 10^7^ cells were washed with PBS once and incubated with ice-cold IP-MS cell lysis buffer on ice for 10 min with periodic mixing. After centrifugation at 13,000×*g* for 10 min, the supernatant was transferred to a new tube for protein quantification. Then, 10 μl of His-tag antibodies (1:100, Cell Signaling Technology), or 2 μg GITRL antibodies (Invitrogen) or 2 μg control IgG (Sigma-Aldrich, St Louis, MO, USA) was incubated with cell lysate at 4 °C overnight. Prewashed magnetic beads were added to the sample/antibody mixture for incubation at room temperature for 1 h with agitation. After collection by the magnetic stand and washing with IP-MS Buffer A three times and IP-MS Buffer B twice, the beads were eluted by IP-MS elution buffer at room temperature for 10 min. Finally, the elution buffer was dried with a speed vacuum concentrator D-AQ (Eppendorf, Germany) for 2 h, and the pulldown proteins were resuspended in protein lysis buffer. After centrifugation at 1200 rpm for 2 min, the supernatant was used for SDS-PAGE analysis, and the protein bands revealed by silver staining were cut out for mass spectrometry analysis (BGI).

### Statistical analysis

Sample sizes were chosen based on previous similar experimental outcomes. Data are presented as the mean value ± SD and were analysed for significance using non-parametric *t* tests by GraphPad Prism 6 software (GraphPad Software, Inc., CA, USA). *P* < 0.05 indicated a significant difference.

## Supplementary information

Supplementary Figure 1

Supplementary Tables

Supplementary movie 1

Supplementary movie 2

Supplementary movie 3

Supplementary movie 4

Author checklist

Declaration of contributions

## Data Availability

RNA array data (GEO accession number: GSE165858) and RNA sequencing data (NCBI Sequence Read Archive accession number: PRJNA698431) used during the study are available online in accordance with funder data-retention policies.
