# Supplementary figures and images for: GITR/GITRL reverse signalling modulates the proliferation of hepatic progenitor cells by recruiting ANXA2 to phosphorylate ERK1/2 and Akt
Source: Cell Death Dis. 2022 Apr 4;13(4):297. doi: 10.1038/s41419-022-04759-z (PMC8979965; doi:10.1038/s41419-022-04759-z)

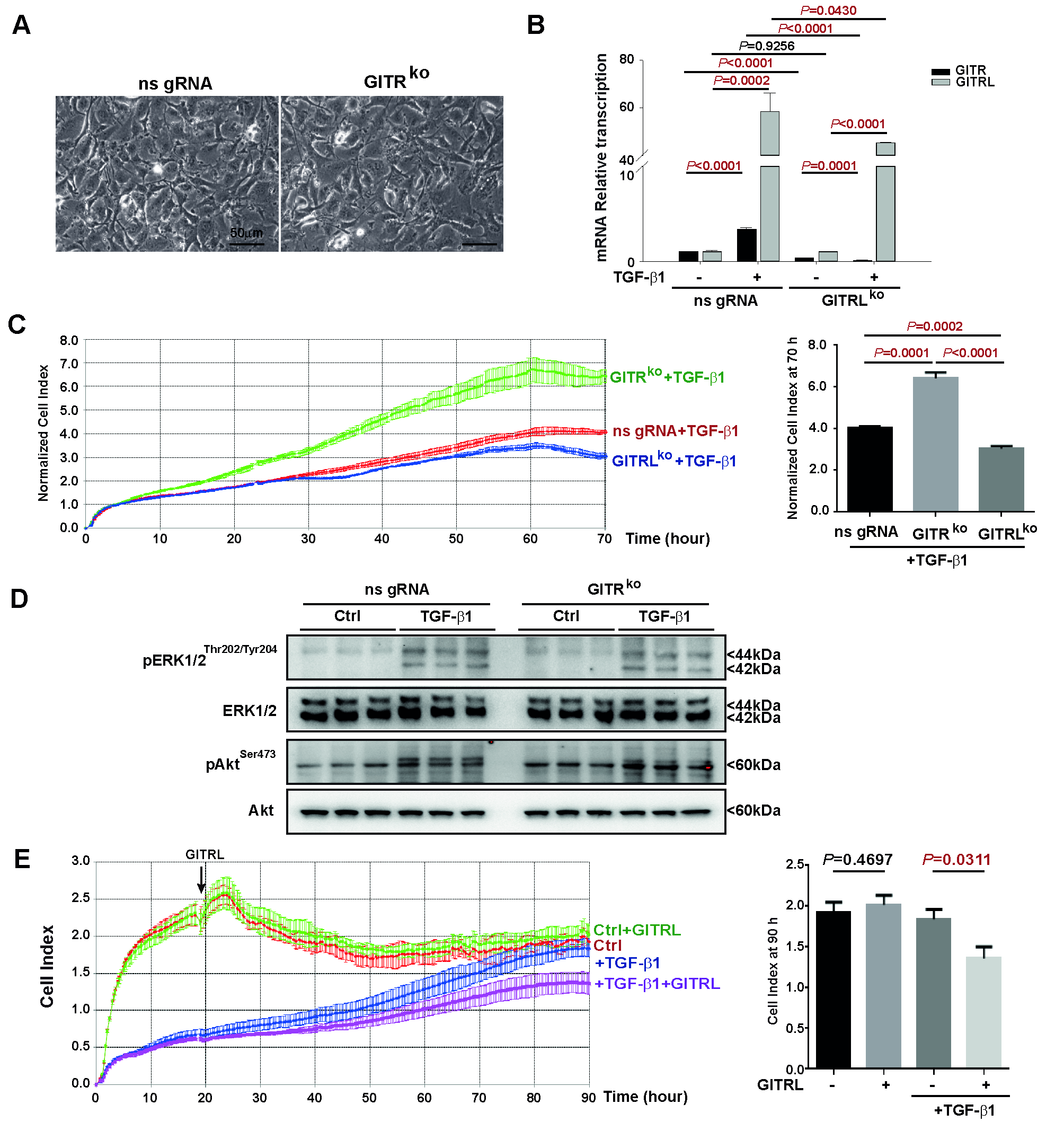

Supplement: Supplementary file 1 — Supplementary Figure 1 [file 41419_2022_4759_MOESM1_ESM.tif]
