# Supplementary material for: GITR/GITRL reverse signalling modulates the proliferation of hepatic progenitor cells by recruiting ANXA2 to phosphorylate ERK1/2 and Akt
Source: Cell Death Dis. 2022 Apr 4;13(4):297. doi: 10.1038/s41419-022-04759-z (PMC8979965; doi:10.1038/s41419-022-04759-z)
Supplement: Supplementary file 2 — Supplementary Tables [file 41419_2022_4759_MOESM2_ESM.docx]

Table S1. TaqMan gene expression assay used in this study.

| Name | Assay-ID | Dye Label |
| --- | --- | --- |
| Tnfsf18(GITRL) | Rn01483793_m1 | FAM |
| Tnfrsf18(GITR) | Rn01515573_m1 | FAM |
| Acta2(αSMA) | Rn01759928_g1 | FAM |
| Anxa2 | Rn00571516_m1 | FAM |
| Gapdh | Rn01775763_g1 | FAM |

Table S2 Clinicopathological characteristics of the tissue sections for histology analysis.

| Case | Age | Sex | HBV | HCV | Diagnosis |
| --- | --- | --- | --- | --- | --- |
| Normal | 38 | M | - | - | - |
| Cirrhotic | 51 | M | + | - | HBV cirrhosis |

Table S3. Antibodies and recombinant proteins used in this study.

| Name | Company | Clone | Catalog No. |
| --- | --- | --- | --- |
| GITRL antibodies | Invitrogen | Polyclone | PA5-47885 |
| GITRL antibodies | ProteinTech | Polyclone | 23899-1-AP |
| GITR antibodies | Abcam | CAL61 | Ab237725 |
| Lgr5 antibodies | OriGene | OTI2A2 | TA503316 |
| Ki-67 antibodies | ProteinTech | Polyclone | 19972-1-AP |
| ERK1/2(Thr202/Tyr204) antibodies | Cell signaling | D13.14.4E | #4370 |
| ERK1/2 antibodies | Cell signaling | 137F5 | #4695 |
| Akt(Ser473) antibodies | Cell signaling | D9E | #4060 |
| Akt antibodies | Cell signaling | 19H8 | #4257 |
| PCNA antibodies | Cell signaling | PC10 | #2586 |
| GAPDH antibodies | ProteinTech | Monoclone | 60004-1-Ig |
| His-Tag antibodies | Cell signaling | D3l10 | #12698 |
| ANXA2 antibodies | Cell signaling | D11G2 | #8235 |
| Rabbit IgG | Sigma-Aldrich | Polyclone | 12-370 |
| Goat IgG | Sigma-Aldrich | Polyclone | NI02-100UG |
| human TGF-β1 | PeproTech | – | 100-21 |
| Rat GITR-Fc Chimera Protein with mouse IgG2A Fc | R&D Systems | – | 9130-GR-050 |
| Mouse IgG2A Fc | R&D Systems | – | 4460-MG-100 |
| Rat GITRL | R&D Systems | – | 9735-GL-050 |
