# Supplementary material for: GITR/GITRL reverse signalling modulates the proliferation of hepatic progenitor cells by recruiting ANXA2 to phosphorylate ERK1/2 and Akt
Source: Cell Death Dis. 2022 Apr 4;13(4):297. doi: 10.1038/s41419-022-04759-z (PMC8979965; doi:10.1038/s41419-022-04759-z)
Supplement: Supplementary file 8 — Declaration of contributions [file 41419_2022_4759_MOESM8_ESM.pdf]

DECLARATION OF CONTRIBUTIONS TO ARTICLE **ADMC**

Manuscript Number: CDDIS-21-1846RR	Journal Name: *Cell Death & Disease*	(the 'Journal')
Proposed Title of the Contribution: GITR/GITRL Reverse Signalling Modulates the Proliferation of Hepatic Progenitor Cells by Recruiting ANXA2 to Phosphorylate ERK1/2 and Akt		(the 'Contribution)
Author(s): Yu He, Yufeng Pei, Kai Liu, Lin Liu, Yue Tian, Hongyi Li, Min Cong, Tianhui Liu, Hong Ma, Hong You, Jidong Jia, Dong Zhang and Ping Wang		(the 'Authors')

For all *CDDis* articles, each person named as an author in the published version must be able to show he or she has contributed substantially to the article.

Authorship credit should be based on 1) substantial contributions to conception and design, acquisition of data, or analysis and interpretation of data; 2) drafting the article or revising it critically for important intellectual content; and 3) final approval of the version to be published. Authors should meet conditions 1, 2 and 3.

Any person who cannot be shown to have made a substantial contribution to the article cannot be listed as an author in the final version. The name of any person who is deemed to have made a minor contribution can, however, appear in the Acknowledgments section of the article.

Please complete the table below to indicate the contributions of all named authors to the manuscript.

Author Full Name:	Specification of Contribution to the Manuscript:
Yu He	did experiment work.
Yufeng Pei	did experiment work.
Kai Liu	did experiment work.
Lin Liu	did experiment work.
Yue Tian	did experiment work.
Hongyi Li	did experiment work.
Min Cong	helped do the experiment work.
Tianhui Liu	helped do the experiment work.
Hong Ma	helped do the experiment work and edit the manuscript.
Hong You	helped do the experiment work and edit the manuscript.
Jidong Jia	designed experiments and edited the manuscript.
Dong Zhang	designed experiments and edited the manuscript.
Ping Wang	designed experiments, did experiment work, and wrote the manuscript.

Please complete the table below to indicate the contributions of all named authors to the figures.

Figure 1:

Yu He and Lin Liu generated the data and prepared panel A and E.
Ping Wang and Yufeng Pei generated the data and prepared panel B, C and D.
Kai Liu generated the data and prepared panel F.
Ping Wang generated the data and prepared panel G.

Figure 2:

Ping Wang generated the data and prepared panel A and B.
Yu He and Hongyi Li generated immune-fluorescence data, labelled the image for panel C, D, E, and F.

Figure 3:

Yu He and Yue Tian generated the data and prepared panel A and G.
Ping Wang generated the data and prepared panel B.
Yu He and Lin Liu generated the data and prepared panel C.
Kai Liu generated the data and prepared panel D.
Yufeng Pei generated the data and prepared panel E and F.
Yu He and Hongyi Li generated the data and prepared panel H.

Figure 4:

Yu He and Lin Liu generated the data and prepared panel A and B.
Kai Liu generated the data and prepared panel C.
Ping Wang generated the data and prepared panel D and E.

Figure 5:

Yufeng Pei generated the data and prepared panel A and B.
Yu He and Min Cong generated the data and prepared panel C.
Yu He and Yue Tian generated the data and prepared panel D.

Figure 6:

Yu He and Tianhui Liu generated the data and prepared panel A.
Yu He and Min Cong generated the data and prepared panel B.
Yu He and Yue Tian generated the data and prepared panel C and E.
Ping Wang generated the data and prepared panel D.
Figure 7
Ping Wang, Hong Ma, and Hong You generated the data and prepared panel A, C and E.
Yu He and Hongyi Li generated the data and prepared panel D.
Kai Liu generated the data and prepared panel B and I.
Yu He and Lin Liu generated the data and prepared panel F, G, H, and J.
Ping Wang, Dong Zhang, and Jidong Jia prepared panel K.

Signed for and on behalf of the Author(s):	Print Name:	Date:
Ping Wang	Ping Wang	2/22/2022
